# Investigating a possible causal relationship between maternal serum urate concentrations and offspring birthweight: a Mendelian randomization study

**DOI:** 10.1093/ije/dyac186

**Published:** 2022-10-03

**Authors:** Caitlin S Decina, Rhian Hopkins, Jack Bowden, Beverly M Shields, Deborah A Lawlor, Nicole M Warrington, David M Evans, Rachel M Freathy, Robin N Beaumont

**Affiliations:** Department of Clinical and Biomedical Sciences, Faculty of Health and Life Sciences, University of Exeter, Exeter, UK; University of Queensland Diamantina Institute, University of Queensland, Brisbane, Queensland, Australia; Institute for Molecular Bioscience, University of Queensland, Brisbane, Queensland, Australia; Department of Clinical and Biomedical Sciences, Faculty of Health and Life Sciences, University of Exeter, Exeter, UK; Department of Clinical and Biomedical Sciences, Faculty of Health and Life Sciences, University of Exeter, Exeter, UK; Department of Clinical and Biomedical Sciences, Faculty of Health and Life Sciences, University of Exeter, Exeter, UK; Medical Research Council Integrative Epidemiology Unit, University of Bristol, Bristol, UK; Population Health Science, Bristol Medical School, University of Bristol, Bristol, UK; Bristol NIHR Biomedical Research Centre, Bristol, UK; University of Queensland Diamantina Institute, University of Queensland, Brisbane, Queensland, Australia; Institute for Molecular Bioscience, University of Queensland, Brisbane, Queensland, Australia; K.G. Jebsen Center for Genetic Epidemiology, Department of Public Health and Nursing, NTNU, Norwegian University of Science and Technology, Trondheim, Norway; University of Queensland Diamantina Institute, University of Queensland, Brisbane, Queensland, Australia; Institute for Molecular Bioscience, University of Queensland, Brisbane, Queensland, Australia; Medical Research Council Integrative Epidemiology Unit, University of Bristol, Bristol, UK; Department of Clinical and Biomedical Sciences, Faculty of Health and Life Sciences, University of Exeter, Exeter, UK; Medical Research Council Integrative Epidemiology Unit, University of Bristol, Bristol, UK; Department of Clinical and Biomedical Sciences, Faculty of Health and Life Sciences, University of Exeter, Exeter, UK

**Keywords:** Birthweight, serum urate, Mendelian randomization, blood pressure, genetics

## Abstract

**Background:**

Higher urate levels are associated with higher systolic blood pressure (SBP) in adults, and in pregnancy with lower offspring birthweight. Mendelian randomization (MR) analyses suggest a causal effect of higher urate on higher SBP and of higher maternal SBP on lower offspring birthweight. If urate causally reduces birthweight, it might confound the effect of SBP on birthweight. We therefore tested for a causal effect of maternal urate on offspring birthweight.

**Methods:**

We tested the association between maternal urate levels and offspring birthweight using multivariable linear regression in the Exeter Family Study of Childhood Health (EFSOCH; *n *=* *872) and UK Biobank (UKB; *n *=* *133 187). We conducted two-sample MR to test for a causal effect of maternal urate [114 single-nucleotide polymorphisms (SNPs); *n *=* *288 649 European ancestry] on offspring birthweight (*n *=* *406 063 European ancestry; maternal SNP effect estimates adjusted for fetal effects). We assessed a causal relationship between urate and SBP using one-sample MR in UKB women (*n *=* *199 768).

**Results:**

Higher maternal urate was associated with lower offspring birthweight with similar confounder-adjusted magnitudes in EFSOCH [22 g lower birthweight per 1-SD higher urate (95% CI: –50, 6); *P *=* *0.13] and UKB [–28 g (95% CI: –31, –25); *P *=* *1.8* × *10^–75^]. The MR causal effect estimate was directionally consistent, but smaller [–11 g (95% CI: –25, 3); *P*_IVW_* *=* *0.11]. In women, higher urate was causally associated with higher SBP [1.7 mmHg higher SBP per 1-SD higher urate (95% CI: 1.4, 2.1); *P *=* *7.8* × *10^–22^], consistent with that previously published in women and men.

**Conclusion:**

The marked attenuation of the MR result of maternal urate on offspring birthweight compared with the multivariable regression result suggests previous observational associations may be confounded. The 95% CIs of the MR result included the null but suggest a possible small effect on birthweight. Maternal urate levels are unlikely to be an important contributor to offspring birthweight.

Key Messages

- Previous research suggests that higher maternal serum urate in pregnancy is associated with lower offspring birthweight and Mendelian randomization studies suggest a causal relationship between urate and systolic blood pressure (SBP), and SBP and birthweight; a causal effect of urate on birthweight has not yet been estimated and thus it is also unknown whether it confounds maternal SBP–birthweight effects.
- The causal effect estimate of urate on offspring birthweight was directionally consistent with, but weaker than, observational estimates; the estimate had 95% CIs that included the null.
- This study confirmed a causal association between serum urate and higher SBP in women, consistent with that published from a sample of both women and men.
- Maternal urate is unlikely to be a major determinant of birthweight or an important confounder of the causal relationship between SBP and lower birthweight.

## Introduction

Lower birthweights are associated with adverse maternal and fetal outcomes in the perinatal period and in later life, most notably with conditions such as type 2 diabetes and cardiovascular diseases.^1^^,^^2^ Various maternal factors are known to be associated with birthweight in observational studies but the causal nature of the associations is not always known. The study of the genetics of cardiometabolic risk factors has led to clearer understanding of some of these associations, such as for systolic blood pressure (SBP), where higher maternal SBP has been causally associated with lower offspring birthweight.^3–5^

Higher maternal serum urate levels are associated with lower offspring birthweight in epidemiological studies^6^^,^^7^ and are associated with higher SBP in pregnancies with low birthweight outcomes.^8^ Raised serum urate levels are associated with cardiovascular diseases such as stroke, hypertension, atherosclerosis and coronary heart disease in the general population^9–12^ and recently Mendelian randomization (MR) and clinical trial data of urate-lowering treatment have suggested that increases in serum urate exert a causal effect on higher SBP.^13^ Although there is an apparent causal relationship between urate and SBP, and SBP and birthweight, a causal effect of urate on birthweight has not yet been estimated.

Observational associations in traditional epidemiological studies are prone to bias, confounding and reverse causality, and thus findings can be misleading. To overcome this, methods such as MR, which uses genetic variants as instrumental variables (IVs) to estimate the causal effect of the exposure of interest on the outcomes, can be applied to observational data.^14^ Genetic variants can be regarded as valid IVs if three key assumptions are met: (i) the IVs are robustly associated with the exposure of interest, (ii) there are no residual confounders between the genetic IVs and outcome, and (iii) the IVs are (potentially) associated with the outcome only through the exposure of interest.^15^ Indeed, MR studies share many similarities with randomized–controlled trials. As alleles randomly segregate and assort independently, variants that proxy exposures should also be independent of environmental influences that might otherwise induce confounding between the exposure and outcome.^14^ Also, because an individual’s germline genotype must precede the outcome of interest, reverse causality is typically not an issue in MR studies. MR studies have recently been applied to examine potential causal relationships between maternal environmental exposures and their offspring’s outcomes.^3–5^ Methods have been developed within this context to enable two-sample MR, i.e. where different samples of individuals are used to estimate the maternal variant–maternal exposure relationship and the maternal variant–offspring outcome relationship.^16^^,^^17^ These methods are useful for incorporating data from large consortia and thus increasing statistical power.^18^^,^^19^

In this study, we aimed to (i) explore associations between maternal serum urate and offspring birthweight in a cohort of pregnant women from the Exeter Family Study of Childhood Health (EFSOCH), as well as with retrospective maternally reported offspring birthweight in women from UK Biobank (UKB); (ii) use MR to test for a causal effect of maternal urate concentration on offspring birthweight; and (iii) use MR to confirm that urate is causally related to SBP in women.

## Methods

### Study overview

**Figure 1:**
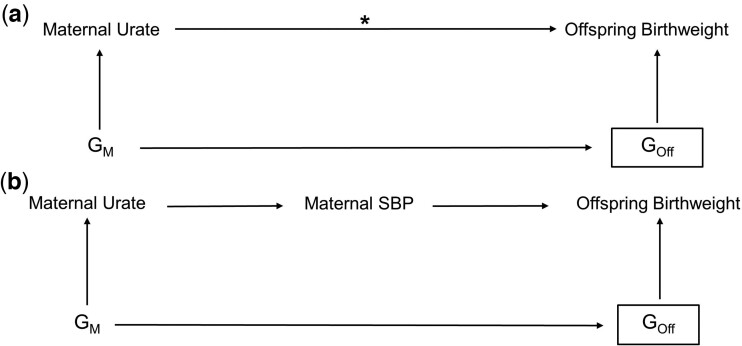
Directed acyclic graph illustrating the plausible (a) or known (b) relationships among key variables [solid arrows; e.g. Mendelian randomization (MR) analyses show a causal effect of higher urate resulting in higher systolic blood pressure (SBP) and higher SBP resulting in lower birthweight] where offspring birthweight is the outcome and maternal urate is the exposure of interest. (a) The putative relationships of the main model we aimed to investigate, estimating the total causal effect of maternal urate on offspring birthweight where the asterisk denotes the causal association being tested. Multivariable observational analyses suggest an inverse effect of higher maternal circulating urate lowering infant birthweight. We used MR with maternal genetic instrumental variables (G_M_) to explore whether there was an inverse total causal effect of urate on birthweight and, if so, whether urate might be a confounder of the SBP–birthweight inverse effect. A rectangular box denotes the variable and its effects have been adjusted for. As offspring genome (G_Off_) has effects on own birthweight, maternal effects were estimated as independent of the offspring genome to capture the maternal genome’s effect on offspring birthweight alone. (b) The known relationships between urate and SBP, and SBP and offspring birthweight. SBP, systolic blood pressure; G_M_, maternal genotype; G_Off_, offspring’s genotype

We first estimated the observational association between maternal serum urate and offspring birthweight in women from both the EFSOCH^20^ and UKB^21^ studies and compared these with previously published associations. We compared our urate–birthweight observational associations with the causal effect estimate obtained from univariate two-sample MR analyses using a set of associated genetic variants, i.e. single-nucleotide polymorphisms (SNPs), as instrumental variables for maternal serum urate. We used estimates for the variant association with the exposure and outcome from the largest, most recent genome-wide association studies (GWAS) available for each variable.^4^^,^^22^ We then tested for a causal relationship between serum urate and SBP in women only by performing one-sample MR in 199 768 women within UKB to compare to recently published findings obtained from a sample of both men and women. All data sets used comprised participants of genetically defined European ancestry to obtain a homogeneous large, well-powered sample. Figure 1 depicts the putative relationships between all variables considered, with the relationship between maternal urate and offspring birthweight being the primary focus of the investigation.

### Observational association of maternal urate with offspring birthweight

We performed multivariable linear regression to explore the association between maternal urate levels measured at 28 weeks’ gestation (exposure of interest) and offspring birthweight (outcome) in a sample of 872 unrelated women within the EFSOCH study. The EFSOCH study is a prospective study of children born between 2000 and 2004, and their parents, from a geographically defined region of Exeter, UK, with participants selected to comprise a homogeneous, European-ancestry cohort. DNA samples and anthropometric measurements were obtained from fasting blood samples (including µmol/L of serum urate) from both parents collected at the study visit when the women were 28 weeks pregnant and offspring DNA was obtained from cord blood at birth, with anthropometric measurements taken at birth, 12 weeks, 1 year and 2 years of age.^20^ Urate levels were determined from blood using a cobas c analyser with a commercially available assay (Roche/Hitachi) that employed an enzymatic, colorimetric method. We included the following as confounders in our regression models on the basis that they are known or plausible causes of variation in urate levels, blood pressure and birthweight: maternal age, height, body mass index (BMI), smoking status, 28-week fasting glucose, estimated glomerular filtration rate (eGFR) and Townsend Deprivation Index (TDI) at pregnancy; additionally we adjusted for child’s sex and gestational age as these are strongly associated with birthweight and may improve model precision. EFSOCH did not have data available for mothers’ pregnancy diet, including alcohol intake or physical activity, which might also confound the associations we are interested in and therefore could not be adjusted for in this model.

The EFSOCH sample provided the benefit of investigating associations using measurements taken during pregnancy, although it was limited in sample size. We therefore also performed observational analyses in a larger sample of UKB women with the limitation that participants’ urate levels were measured after pregnancy. UKB is a large prospective cohort study of 500 000 participants (5.5% of those invited) assessed throughout the UK between 2006 and 2010, 40–69 years of age at baseline. UKB includes a wide range of phenotypic and genotypic data collected from self-completed questionnaire, interview, physical and functional measures, and collection of blood, urine and saliva.^21^ Urate levels specifically, were measured by uricase PAP analysis on a Beckman Coulter AU5800.^23^ A multivariable linear regression model was used to explore the association between maternal urate levels measured on average 31.6 (SD = 10.1) years after delivery and offspring birthweight among 133 187 unrelated women within UKB who reported the birthweight of their first child. This model was also adjusted for potential confounding by maternal age at study enrolment (when urate was measured), maternal age at first birth, height, BMI, smoking status, alcohol intake, eGFR, blood glucose level, TDI and assessment centre location. Information on child’s sex and gestational age at delivery were not available in UKB and therefore were not adjusted for in this analysis.

### Genetic instruments for maternal serum urate concentration

The genetic instruments used for maternal serum urate in the MR analysis were genetic variants identified in a recently published trans-ancestry GWAS of serum urate in 457 690 individuals across 74 studies where UKB was not used for discovery analysis of urate-associated variants.^22^ In addition to the trans-ancestry GWAS, Tin *et al.* conducted a meta-analysis and subsequent fine-mapping using the subset of studies of European descent (60 studies including 288 649 participants excluding UKB), identifying 114 independent SNPs at genome-wide significance (*P *≤* *5 × 10^−8^) (Supplementary Table S1, available as Supplementary data at *IJE* online). Variants were annotated using NCBI b37 (hg19). Preparation of phenotype data was standardized across the included studies using a common script and study-specific association analyses were uniform across all studies through the following of a centrally developed analysis plan. More information on study-specific analyses can be found in Tin *et al*.^22^ We calculated the proportion of variance explained by each of the 114 SNPs based on the following formula:

$$\beta^{2}\left(\frac{2p(1-p)}{var(Y)}\right)$$

where *β* is the effect size estimate for the SNP, *p* is the minor allele frequency for the SNP and *var*(*Y*) is the phenotypic variance. We used the phenotypic variance of 1.767 as reported by Tin *et al.*^22^ from the Atherosclerosis Risk in Communities study. As each of the 114 SNPs is independent, we summed the proportion of variance explained by each SNP and found that the 114 SNPs explain 6.4% of the variance in urate. We assessed the validity of these instruments with respect to strength and amount of heterogeneity via calculation of a mean F statistic^24^ = 126.9 and Cochran’s Q statistic^25^ = 257.1, respectively.

### Validating genetic instrument for urate in pregnant women

As the urate-associated SNPs used in this study were identified in a sample of males and non-pregnant females, we wanted to determine whether the genetic instruments were good predictors of urate levels in pregnancy as well as outside of pregnancy. Therefore, we created a weighted, standardized genetic score (GS) for urate and tested its association with urate levels in women in the EFSOCH study where urate was measured in participants in both periods: at 28 weeks’ gestation (*n *=* *872) and approximately 6 years post-pregnancy (*n *=* *470).^20^ The genotyping and imputation methods for EFSOCH mothers and babies have been described previously.^26^ The GS was calculated according to Equation (1):

(1) $$GS=\;\sum_{i}w_{i}g_{i}$$

where w_*i*_ is the weight for SNP *i* and g_*i*_ is the genotype (number of effect alleles 0–2) at SNP *i*. The SNP weightings were the regression coefficients obtained from the most recently reported GWAS of urate as mentioned above.^22^ The calculated GS was then standardized and the urate levels for participants (µmol/L) in each time period regressed on the standardized genetic score.

Of the 114 urate-associated SNPs, 10 did not have genotype data available in EFSOCH. For these 10 SNPs, we searched for suitable proxies using the LDproxy tool.^27^ SNPs found with the highest *r*^2^ (minimum *r*^2^ = 0.8) in a European population within a 500-kbp window of the target SNP were selected for analysis. Four SNPs did not have proxies available that met the *r*^2^ threshold and were excluded from analysis; therefore, a total of 110 urate-associated SNPs were used to compose the GS (Supplementary Table S2, available as Supplementary data at *IJE* online).

### MR of maternal urate on offspring birthweight

We used two-sample MR to test for a causal association between maternal urate and offspring birthweight. The variant–exposure associations were obtained using the European-ancestry-specific summary data from the serum urate GWAS mentioned previously (Sample 1).^22^ The variant–outcome associations were obtained from a GWAS of own birthweight and offspring birthweight in a total of 406 063 European-ancestry individuals from the Early Growth Genetics (EGG) Consortium and UKB, where maternal and fetal genetic effects on birthweight (i.e. the indirect effect of a mother’s genotype on offspring birthweight through the intrauterine environment and the direct effect of an individual’s genotype on their own birthweight, respectively) were estimated using a weighted linear model (WLM) (Sample 2).^4^ The weighted linear model developed by Warrington *et al.* provides unbiased estimates of the maternal-specific and fetal-specific genetic effects on birthweight by accounting for the correlation between fetal and maternal genotypes.^4^ Using the maternal-specific effects obtained by this method ensures that we are not violating the exclusion restriction MR assumption due to confounding from the fetal genotype. The total number of participants comprised 101 541 UKB participants who reported their own birthweight and the birthweight of their first child, 195 815 UKB and EGG participants with own birthweight data, and 108 707 UKB and EGG participants with offspring birthweight data.

Inverse-variance weighted (IVW; fixed effects) MR was performed along with additional pleiotropy-robust sensitivity analyses, including MR–Egger, weighted median and penalized weighted median estimators. The IVW method of estimating causal effects regresses the summary association of each SNP with outcome against the summary association with exposure, constraining the regression line to pass through a zero intercept. It thus assumes that there is no bias due to unbalanced horizontal pleiotropy^28^ whereas MR–Egger is identical to IVW except it fits the best regression line without constraining it through zero and thus provides a means to test and correct for bias in the IVW estimate when variants affect the outcome through pathways other than the exposure of interest (i.e. unbalanced horizontal pleiotropy).^29^ MR–Egger provides a consistent causal estimate under the assumption that the association of each genetic variant with the exposure is independent of the pleiotropic effect of the variant (i.e. pleiotropic effects are not proportional to the variants’ effects on the exposure of interest) even when all of the variants are invalid instruments.^29^ A non-null intercept value in MR–Egger provides statistical support for the IVW results being biased by unbalanced horizontal pleiotropy. Weighted median MR additionally provides a consistent estimate when at least half of the variance explained in the genetically predicted exposure comes from valid IVs (not pleiotropic).^30^ It is less prone to the effects of SNPs with outlier effects than IVW or MR–Egger, with penalization providing added robustness to avoid effects of large outliers. Details of the R code for the MR analyses are provided elsewhere.^29^^,^^30^

### Assessing specific potential horizontal pleiotropic paths

In addition to a general approach to exploring potential bias due to unbalanced horizontal pleiotropy using MR–Egger and weighted medians, we explored the possibility of results being influenced by specific pleiotropic paths by examining the association of our genetic instruments for urate with maternal smoking behaviour. Specifically, we checked that our selected urate variants were independent of smoking behaviour by looking up the 114 variants in a recent GWAS of risky behaviours including smoking^31^ to determine how many SNPs were associated with smoking behaviour at genome-wide significance (*P *≤* *5* × *10^–8^) and at a Bonferroni-corrected *P*-value of *P *≤* *4.4* × *10^–4^ for the number of SNPs being tested. We also tested the association between the urate GS composed of all 114 SNPs described previously and smoking in a sample of 918 EFSOCH women who reported they smoked during pregnancy.

### Validating a causal effect of urate on systolic blood pressure

We sought to confirm a causal relationship between serum urate and SBP in UKB women, our main population of interest, as previous causal findings used a mixed sex sample excluding UKB participants.^13^ We thus performed one-sample MR in 199 768 women within the UKB using two-stage least squares, with estimation conducted using the ivreg2 function in Stata version 16.0.^32^^,^^33^ This analysis was instrumented by a GS using the same 114 urate-associated SNPs from our main MR analysis and constructed for the UKB women in the same manner as described above. We did not adjust for any covariates.

## Results

### Observational associations

Baseline characteristics of the EFSOCH and UKB women included in each multivariable linear regression model are described in Tables 1 and 2, respectively.

**Table 1:** Descriptive characteristics of Exeter Family Study of Childhood Health women included in the multivariable linear model of factors associated with child’s birthweight, both in pregnancy and post-partum

Characteristic	Mean (SD) for continuously measured variables and *n* (%) for categorical variables	
	In pregnancy (*n *=* *872)	Post-pregnancy (*n *=* *470)
Child’s gestational age at delivery (weeks)	40.1 (1.2)	40.1 (1.2)
Child’s sex^a^		
Male	448 (51.4)	236 (50.2)
Female	424 (48.6)	234 (49.8)
Child’s birthweight (g)	3503.0 (476.8)	3471.0 (494.7)
Age (years)	30.5 (5.1)	36.7 (5.0)
Body mass index (kg/m^2^)	27.9 (4.6)	24.8 (4.4)
Mean height (cm)	165.0 (6.3)	165.1 (6.4)
Serum urate (µmsssol/L)	214.3 (41.2)	254.6 (50.3)
Smoking status^a^		
Never	623 (71.4)	354 (77.0)
Former	128 (14.7)	59 (12.8)
Current	121 (13.9)	47 (10.2)
Townsend Deprivation Index^b^	0.3 (3.3)	0.07 (3.3)
Fasting glucose (mmol/L)	4.3 (0.4)	4.6 (0.5)
eGFR (mL/min/1.73 m^2^)	116.7 (11.3)	98.1 (13.8)

Measurements during pregnancy were taken at 28 weeks’ gestation.

a Categorical variables for which data are presented as count (%) rather than mean (SD).

b The Townsend Index is a measure of material deprivation in a population. A score is calculated using four variables obtained from census data: (i) percentage of people unemployed, (ii) percentage of non-car owners, (iii) percentage of households not owner occupied and (iv) percentage of households overcrowded. Positive scores represent more deprivation and negative scores represent more wealth.^46^

eGFR, estimated glomerular filtration rate.

**Table 2:** Baseline characteristics of UK Biobank women included in the multivariable model of factors associated with first child’s birthweight (*n *=* *133 187)

Characteristic	Mean (SD) for continuously measured variables and *n* (%) for categorical variables
Child’s birthweight (g)	3227.8 (477.1)
Age at enrolment (years)	57.7 (7.7)
Age at first birth (years)	26.0 (5.0)
Body mass index (kg/m^2^)	27.0 (5.0)
Height (cm)	162.5 (6.1)
Serum urate (µmol/L)	270.8 (65.4)
Smoking status^a^	
Never	78 214 (58.7)
Former	44 337 (33.3)
Current	10 636 (8.0)
Alcohol intake frequency^a^	
Daily or almost daily	22 143 (16.6)
3 or 4 times/week	28 766 (21.6)
1 or 2 times/week	35 594 (26.7)
1–3 times/month	17 321 (13.0)
Special occasions only	18 928 (14.2)
Never	10 435 (7.8)
Townsend Deprivation Index	–1.7 (2.8)
Blood glucose (mmol/L)	5.0 (0.7)
eGFR (mL/min/1.73 m^2^)	89.9 (12.9)

a Categorical variables for which data are presented as count (%) rather than mean (SD).

eGFR, estimated glomerular filtration rate.

**Figure 2:**
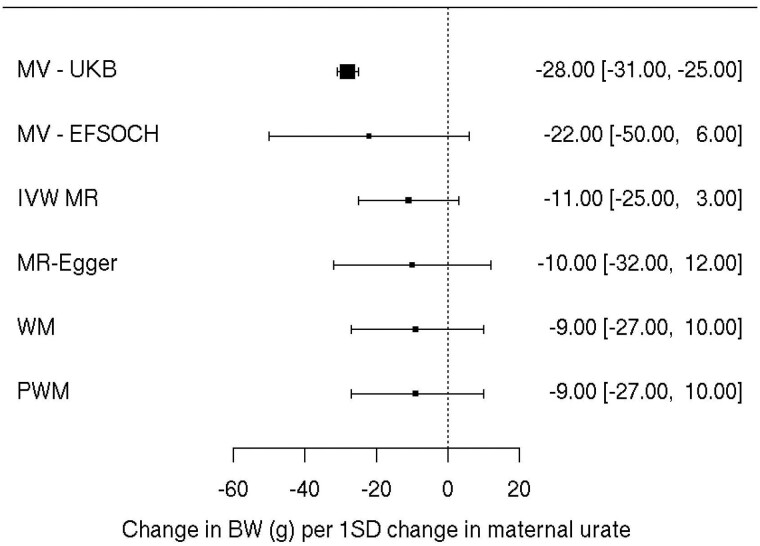
Forest plot of the effect of maternal serum urate on offspring birthweight: observationally using participants in UK Biobank (*n *=* *133 187), observationally using participants in the Exeter Family Study of Childhood Health (*n *=* *872) and using univariate Mendelian randomization (inverse-variance weighted method) with additional sensitivity methods. Each estimate is the change in birthweight in grams per 1-SD change in urate level (95% CI). The MR–Egger analysis indicated no sign of horizontal pleiotropy with an intercept near zero [–0.07 (95% CI: –0.22, 0.08)]. The sizes of the black boxes are a reflection of the sample size for each analysis—the larger the box, the greater the sample size, with the bracketing segments representing the 95% CIs. MV, multivariable linear regression; UKB, UK Biobank; EFSOCH, Exeter Family Study of Childhood Health; IVW, inverse-variance weighted; MR, Mendelian randomization; WM, weighted median; PWM, penalized weighted median; BW, birthweight

Multivariable modelling using the sample of EFSOCH women indicated there was weak evidence of an association between maternal urate levels and offspring birthweight, with a wide 95% confidence interval due to a relatively small sample size [22 g lower birthweight per 1-SD higher maternal urate (95% CI: –50, 6); *P *=* *0.13; Figure 2 and Supplementary Table S3, available as Supplementary data at *IJE* online].

The same associations were then investigated in a much larger sample of UKB women with the caveat of urate having been measured at a mean of 31.6 (SD = 10.1) years post-pregnancy. Here, results indicated that higher maternal serum urate concentration was associated with a lower offspring birthweight [28 g lower birthweight of first child per 1-SD higher maternal urate (95% CI: –31, –25); *P *=* *1.8* × *10^–75^], consistent with that found in EFSOCH (Figure 2 and Supplementary Table S4, available as Supplementary data at *IJE* online).

### Association between urate GS and urate measured during and post-pregnancy

**Figure 3:**
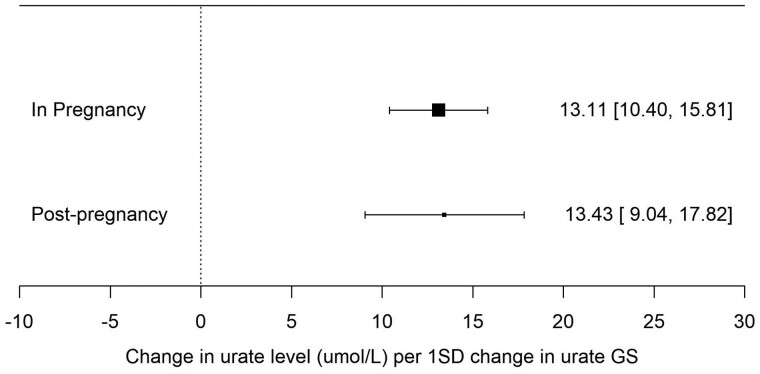
Forest plot of the association between a 110-SNP urate genetic score and urate levels measured in pregnancy (*n *=* *872) and post-pregnancy (*n *=* *470) in the Exeter Family Study of Childhood Health. Each estimate is the change in urate level (µmol/L) per 1-SD change in urate genetic score (95% CI). The sizes of the black boxes are a reflection of the sample size for each analysis—the larger the box, the greater the sample size, with the bracketing segments representing the 95% CIs. GS, genetic score

In the sample of EFSOCH women, the GS for urate showed similar association with urate levels measured in pregnancy at an average maternal age of 30.3 (SD = 5.3) years compared with post-pregnancy measured at an average maternal age of 36.7 (SD = 5.0) years [13.1 µmol/L (95% CI: 10.4, 15.8) and 13.4 µmol/L (95% CI: 9.0, 17.8) higher urate per 1-SD higher urate GS, respectively], indicating that the SNPs associated with urate levels in the general population used for the MR analyses are similarly associated with urate levels in a population of pregnant women (Figure 3).

### Two-sample MR

We did not find strong evidence for a causal effect of maternal urate level on offspring birthweight, although the causal effect estimate for urate was directionally consistent with the observational estimate [11 g lower birthweight per 1-SD higher urate (95% CI: –25, 3); *P*_IVW_ = 0.11]. Sensitivity analyses showed similar results, with the MR–Egger analysis showing no evidence of horizontal pleiotropy as indicated by an intercept of approximately zero [–0.07 (95% CI: –0.22, 0.08)] (Figure 2 and Supplementary Figure S1, available as Supplementary data at *IJE* online).

Two of the 114 GWAS associated urate SNPs were also genome-wide significantly associated with smoking in the latest smoking GWAS,^31^ with an additional six SNPs identified at a Bonferroni-corrected *P*-value of *P *≤* *4.4* × *10^–4^. Five out of eight of those SNPs had effect estimates working in the same direction as the urate-raising allele whereas the other three SNPs had effects in the opposite direction. When excluding all eight SNPs in a sensitivity analysis, the MR causal effect estimate did not meaningfully change [9 g lower birthweight per 1-SD change in maternal urate (95% CI: –22, 4); *P *=* *0.17]. We found no strong evidence of association between our urate GS and smoking behaviour in EFSOCH women who smoked during pregnancy [0.14 SDs higher urate GS if woman smoked during pregnancy (95% CI: –0.05, 0.33); *P *=* *0.14].

### Causal effect of urate on systolic blood pressure

Within UKB women, we found strong evidence for a causal effect of serum urate levels on SBP [1.7 mmHg higher SBP per 1-SD higher serum urate (95% CI: 1.4, 2.1); *P *=* *7.8* × *10^–22^]. Based on 1-SD of SBP = 24.23 mmHg, this is equivalent to 0.07 SD higher SBP per 1-SD higher urate.

## Discussion

In our own observational analyses we found 22 g lower birthweight in the EFSOCH study and 28 g lower birthweight in the UKB per 1-SD higher maternal urate in confounder-adjusted multivariable regression analyses. This is consistent with previous observational studies which have found that higher maternal urate levels show an association with lower offspring birthweight.^6^^,^^7^ When investigating a causal association of maternal urate on offspring birthweight using MR, the effect estimate was directionally consistent with the observational estimates, although imprecise and including the null. The association between maternal SBP and lower birthweight has previously been established as causal, e.g. by Tyrrell *et al.* and Warrington *et al.*^3^^,^^4^ Recently published MR and clinical trial data have now provided evidence of a causal effect of serum urate on SBP.^13^ When investigating this causal relationship in UKB women only, our result was consistent with their findings (0.07 vs 0.09 SD units of SBP per 1-SD higher serum urate, respectively).

**Figure 4:**
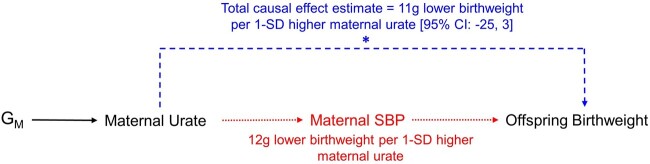
Schematic illustrating the proposed relationships among key variables given the results of this study where offspring birthweight is the outcome, maternal urate is the exposure of interest and maternal systolic blood pressure (SBP) is a mediator. Using Mendelian randomization (MR) we estimated the total causal effect of maternal urate on offspring birthweight, denoted by the starred, dashed line pathway in blue [11 g lower birthweight per 1-SD higher maternal urate (95% CI: –25, 3)]. We subsequently informally estimated ∼12 g lower birthweight per 1-SD higher maternal urate concentration if all of the effect of urate was mediated by SBP, denoted by the dotted pathway in red. As the causal effect estimate of 11 g lower birthweight and the estimated SBP-mediated effect of 12 g lower birthweight are congruent, this suggests no direct effect of maternal urate levels on offspring birthweight and consequently lack of evidence for maternal urate as a potential source of confounding on the existing causal relationship between maternal SBP and offspring birthweight. However, as the span of 95% CIs of the MR analysis are not centred on the null, a true small direct causal effect cannot be ruled out. G_M_, maternal genotype; SBP, systolic blood pressure

Given the estimated causal effect of urate on SBP found here in UKB women, we would correspondingly expect ∼12 g lower birthweight per 1-SD higher maternal urate concentration if all of the effect of urate was mediated by SBP (Supplementary note, available as Supplementary data at *IJE* online). This estimated difference in birthweight sits within the 95% CIs of the urate on birthweight MR causal effect estimate and is indeed very close to the effect estimate itself of ∼11 g lower birthweight per 1-SD change in urate. Thus, our findings currently suggest that urate has at most a modest effect on birthweight that is fully mediated by SBP and therefore urate is unlikely to be a key confounder of the maternal SBP–offspring birthweight effect (Figure 4). However, as the span of 95% CIs of the MR analyses are not centred on the null, a true small direct causal effect cannot be ruled out and thus some confounding by maternal urate on the existing SBP–birthweight effect is possible and may be illuminated by future studies. A formal mediation analysis to assess the effects of SBP on the urate–birthweight relationship would be recommended if subsequent analyses with larger samples provide robust evidence of a causal effect.

Serum urate, or uric acid, is thought to be involved in the biological processes of oxidative stress and inflammation, with particular action in endothelial cells causing vascular damage—mechanisms that may explain its role in increasing SBP and the development of cardiovascular diseases.^34^^,^^35^ With little evidence to currently support a direct causal role for maternal urate lowering the mean offspring birthweight, it is possible that higher mean urate levels seen in pregnancies with small babies reflect the impact of higher urate on blood pressure and of higher blood pressure on lower birthweight. Maternal pre-existing high blood pressure and hypertensive disorders of pregnancy are related to placental vascular malperfusion and dysfunction, which may then exert greater impact on fetal growth than urate might influence placental function through direct mechanisms.^36^ A pathogenic role for urate has also been suggested in the development of pre-eclampsia^37–39^—a hypertensive disorder of pregnancy characterized by the onset of high blood pressure and substantial proteinuria at ≥20 weeks gestation with which high urate levels and low offspring birthweight outcomes are commonly associated.^40–42^ The finding in the current study of no strong evidence for a causal effect of maternal urate level on offspring birthweight is therefore not entirely unexpected given that other circulatory system-related factors such as SBP and abnormal vascular changes in the placenta seen in pre-eclampsia cases have already been shown to play a role in low birthweight outcomes.^3^^,^^36^^,^^43^ Investigating the role of maternal urate in pre-eclampsia using causal methods indeed presents an avenue for future research and an opportunity to provide further clarity on how urate may influence birthweight along a causal pathway.

There are some limitations worth noting in this study. The validity of the observational analysis of the association between maternal urate and offspring birthweight in UKB was limited by participants having urate measurements that were collected several years after pregnancy. However, we used the observational association in UKB to complement the main observational analysis in pregnant women and babies from the EFSOCH study because UKB is larger than EFSOCH, enabling greater precision of estimates. We found consistent estimates in both samples. Whilst we made extensive adjustments for observed confounders, we were not able to adjust for maternal pregnancy diet, alcohol intake or physical activity that might confound the association of urate with birthweight. As noted below, the consistency of our results with previous observational studies together with our MR findings does suggest that our (and previous) observational studies are influenced by residual confounding.

Additionally, there is a small overlap between the cohorts that contributed to the GWAS of serum urate and the EGG consortium GWAS of own (fetal) birthweight but not with the maternal GWAS of offspring birthweight. With respect to impact this might have for overlap between SNP–exposure estimates and SNP–outcome estimates in our study, there will be some influence on the WLM estimates via the fetal effect estimates that are included but not via the maternal estimates and therefore only ∼50% overlap. Together, these cohorts make up a very small proportion of the fetal GWAS sample (*n *=* *5766/321 223; 1.7%); thus, any risk of bias from inclusion of these studies will be minimal.

The SNP–birthweight effect estimates used in our analyses were also mostly unadjusted for gestational duration.^4^ Evidence in the literature is mixed on whether maternal SBP affects fetal growth directly or whether the birthweight effect is mediated by an effect of maternal SBP on gestational duration.^39^ Previous causal MR analyses suggest higher SBP has a direct effect on lower birthweight;^3^^,^^4^ however, recent work suggests that increased maternal SBP is causally associated with shorter gestational duration.^45^ As the birthweight associations used in this study are thus not fully adjusted for gestational age at delivery, we are aware that the effects on birthweight being tested here could reflect a combination of factors, including fetal growth rates or gestational duration or both.

Whilst there was strong evidence of association between a number of urate SNPs used as genetic instruments in this study and smoking behaviour, effects varied in their direction of association with urate and any pleiotropy is therefore potentially biased. However, in a sensitivity analysis excluding the SNPs associated with smoking, the causal effect estimate was largely unchanged. MR–Egger and weighted median analyses also suggested that our results were not substantially biased by unbalanced horizontal pleiotropy as they provided causal effect estimates that were consistent with the main IVW estimate. Importantly we did not see strong evidence of a relationship between a urate GS including these smoking-associated SNPs and maternal urate levels in pregnancy.

As mentioned previously, we used the currently most well-powered sample available to investigate a causal relationship between maternal urate and birthweight; however, our conclusions may still be limited by sample size. Whereas our results suggest that a large causal effect of urate on birthweight is unlikely, they do not rule out smaller effects. Further investigation will allow greater precision in effect estimates and thus to determine whether small but potentially biologically meaningful effects exist, especially in cases such as pre-eclampsia where the impact and role of urate is not confirmed and where small changes could influence whether a pregnancy is classified as higher-risk.

In conclusion, there was a lack of strong evidence for a causal effect of maternal urate on offspring birthweight although 95% CIs suggest the possibility of weak influence that larger studies may provide a more precise estimate of in the future. Stronger associations seen in previously published observational studies may be confounded by other maternal physiological factors such as the effect of SBP. Current evidence indicates that maternal SBP mediates any effect of urate on birthweight and that urate is unlikely to confound the causal effect of maternal SBP on offspring birthweight. Further research into the relationship between maternal urate and pre-eclampsia using causal methods could help to further clarify a role for urate in pregnancy.

## Ethics approval

This study was conducted using the UK Biobank resource. Details of patient and public involvement in UK Biobank are available online (www.ukbiobank.ac.uk/about-biobank-uk/ and https://www.ukbiobank.ac.uk/wp-content/uploads/2011/07/Summary-EGF-consultation.pdf?phpMyAdmin=trmKQlYdjjnQIgJ%2CfAzikMhEnx6). No patients were specifically involved in setting the research question or the outcome measures, nor were they involved in developing plans for recruitment, design or implementation of this study. No patients were asked to advise on interpretation or writing up of results. There are no specific plans to disseminate the results of the research to study participants but UK Biobank disseminates key findings from projects on its website. Ethical approval for EFSOCH was given by the North and East Devon (UK) Local Research Ethics Committee (approval number 1104) and informed consent was obtained from the parents of the newborns. The EGG consortium birthweight GWAS summary data used in this study did not require prior approval for access and are freely available online.

## Supplementary Material

dyac186_Supplementary_Data

## Data Availability

The genotype and phenotype data are available on application from UK Biobank (http://www.ukbiobank.ac.uk/). Individual cohorts participating in the EGG consortium should be contacted directly as each cohort has different data-access policies. GWAS summary statistics of birthweight are available via the EGG website (https://egg-consortium.org/). Genome-wide summary statistics for urate used in this study are publicly available at the CKDGen Consortium via http://ckdgen.imbi.uni-freiburg.de. Summary statistics from EFSOCH are available on request. Researchers interested in accessing the data are expected to send a reasonable request by sending an e-mail to the Exeter Clinical Research Facility at crf@exeter.ac.uk.
